# Pharmacological Activities of Ginkgolic Acids in Relation to Autophagy

**DOI:** 10.3390/ph15121469

**Published:** 2022-11-26

**Authors:** Yuan Ding, Zheheng Ding, Jin Xu, Yueying Li, Min Chen

**Affiliations:** 1 Lab of Clinical Chemistry, Danyang Hospital of Tranditional Medicine, Danyang 212300, China; 2 School of Medicine, Heinrich-Heine University, Moorenstr. 5, 40225 Düsseldorf, Germany; 3 School of Medicine, Jiangsu University, #301 XueFu Road, Zhenjiang 212013, China

**Keywords:** ginkgolic acids, anticancer, neuroprotection, apoptosis, SUMOylation, autophagy

## Abstract

Plant-derived natural compounds are widely used as alternative medicine in healthcare throughout the world. Ginkgolic acids, the phenolic compounds isolated from the leaves and seeds of *Ginkgo biloba*, are among the chemicals that have been explored the most. Ginkgolic acids exhibit cytotoxic activity against a vast number of human cancers in various preclinical models in vitro and in vivo. Additionally, the pharmacological activities of ginkgolic acids are also involved in antidiabetic, anti-bacteria, anti-virus, anti-fibrosis, and reno/neuroprotection. Autophagy as a highly conserved self-cleaning process that plays a crucial role in maintaining cellular and tissue homeostasis and has been proven to serve as a protective mechanism in the pathogenesis of many diseases, including neurodegenerative diseases, cancer, and infectious diseases. In this review, we surveyed the pharmacological activities of the major three forms of ginkgolic acids (C13:0, C15:1, and C17:1) that are linked to autophagic activity and the mechanisms to which these compounds may participate. A growing body of studies in last decade suggests that ginkgolic acids may represent promising chemical compounds in future drug development and an alternative remedy in humans.

## 1. Introduction

Botanical natural products from plants in the form of traditional medicines have been widely used in healthcare worldwide. *Ginkgo biloba* L. (English name: Maidenhair tree), also known as ginkgo, is the only living species in the family Ginkgoaceae [1,2,3]. This wild species native to China is believed to at one point have covered the hill country along the Yangtze River valley border, but is cultivated throughout China and the world [4,5]. This ancient, deciduous, tall, and dioecious reproduction species has very conservative morphology and remarkable genetic stability [6]. Therefore, *Ginkgo biloba* L. is considered as a “living fossil”, because it is one of the oldest seed plants and highly resistant to environmental stresses, microorganisms (bacteria, virus, or fungi), insects, and chemical pollutants [7].

**Figure 1:**
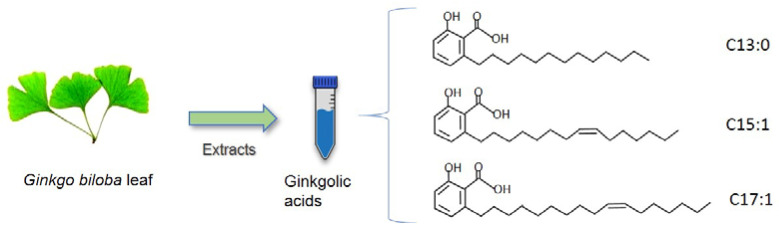
Chemical structures of major ginkgolic acids C13:0, C15:1, and C17:1.

Since thousands of years, the leaves and seeds of *Ginkgo biloba* have been used in Chinese traditional medicine and in other parts of the world as herb supplements. Due to its long history of use in traditional medicine, *Ginkgo biloba* is considered a natural reservoir of molecules with health-promoting potential. *Ginkgo biloba* extracts are the mixture of numerous components, such as flavonoids, terpenoids, trilactones, and a group of alkylphenols (anacardic or ginkgolic acids, cardanols, and cardols) that have been considered as effective chemicals linked to traditional Chinese medicine. The phenolic compounds, ginkgolic acids, are chemically 2-hydroxy-6- alkylsalicylic acids with the common alkyl chains containing 13, 15, or 17 carbons and unsaturated at positions 8 and 10 in the 15 and 17 carbon chains, respectively [8]. C13:0, C15:1, and C17:1, whose structures are designated according to their alkyl chain carbon number (Figure 1), are the major three forms of ginkgolic acids that are most intensively investigated regarding pharmacological and therapeutic studies.

In the last decade, a growing body of studies has indicated an anticancer property of ginkgolic acids, which, together with its other pharmacological roles, suggests that ginkgolic acids may be promising for drug development or in combination with chemotherapeutic drugs against human diseases. In this review, we prospectively survey the pharmacological activities of the three major forms of ginkgolic acids, i.e., C13:0, C15:1, and C17:1, as well as the underlying mechanisms to which they may be attributed, and highlight the autophagic activities that ginkgolic acids may exhibit.

## 2. Pharmacological Effects of Ginkgolic Acids

Recent studies indicate that ginkgolic acids exhibit a wide range of pharmacological activities, which lead to various therapeutic potentials of antidiabetic, anticancer, anti-fibrosis, anti-bacteria, anti-virus, and reno/neuroprotective. We summarize and discuss the individual features as listed below. 

### 2.1. Antidiabetics

Diabetes is a chronic illness characterized by elevated blood glucose levels due to cellular resistance to insulin, insufficient insulin production by pancreatic β-cells, or both. The most common form of diabetes is type 2 diabetes [9], which can affect multiple organs and thus lead to clinical complications including nephropathy, retinopathy, neuropathy, and cardiovascular diseases.

AMP-activated protein kinase (AMPK) is an enzyme known as a master regulator of glycolic metabolism. The activation of the evolutionarily conserved serine/threonine kinase elicits insulin-sensitizing effects, making it an important target for the treatment of type 2 diabetes [10]. The activity of AMPK is negatively regulated by the tyrosine–protein phosphatase non-receptor type 9 (PTPN9), also known as PTP-MEG2, by reducing AMPK degradation. The inhibition of PTPN9, which indirectly enhances AMPK activity in diabetic mice, restores insulin sensitivity and glucose homeostasis [11]. Another phosphatase family member, dual-specificity phosphatase-9 (DUSP-9), also known as MKP4, is also proven to be involved in insulin resistance due to its expression in insulin-sensitive tissues and the modulation of DUSP-9 expression in insulin-resistant states [12].

**Figure 2:**
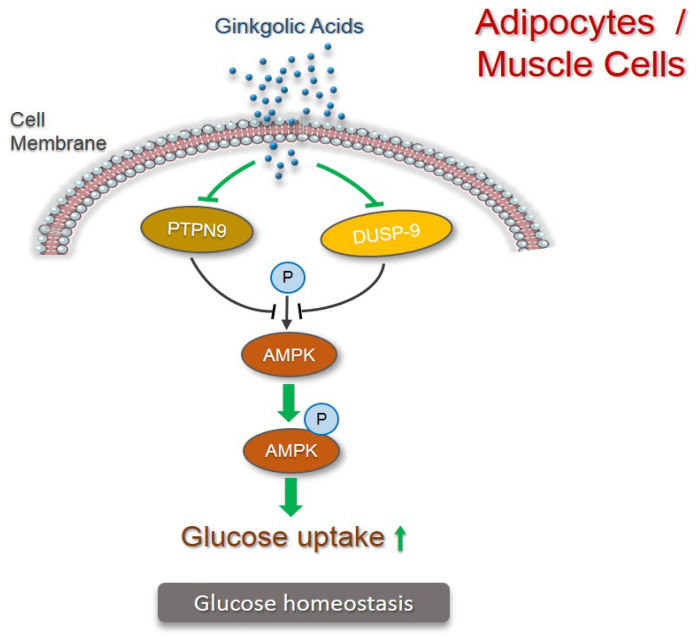
Mechanism for antidiabetics by ginkgolic acids in adipocytes and muscle cells. PTPN9 and DUSP9 negatively regulate the phosphorylation of AMPK (illustrated in black). Ginkgolic acids inhibit activity of PTPN9 and DUSP9, thus increasing phosphorylation of AMPK to increase glucose uptake (illustrated in green).

The knockdown of either PTPN9 or DUSP9 in adipocytes in vitro promoted AMPK phosphorylation, and the concurrent silence of both PTPN9 and DUSP9 expression synergistically enhanced AMPK activity. Notably, the administration of ginkgolic acid (C13:0) increased the phosphorylation of AMPK in 3T3-L1 adipocytes and inhibited the activity of PTPN9 and DUSP9 in differentiated C2C12 muscle cells and 3T3-L1 adipocytes, leading to a significant increase in glucose uptake [13]. These in vitro results suggest that the mechanistic action of ginkgolic acid may function through its inhibitory role on both PTPN9 and DUSP9, which, by increasing the bioactivity of AMPK, increases glucose uptake (Figure 2). Thus, the findings hold the promise of using ginkgolic acid as an AMPK-mediated antidiabetic treatment. However, further studies such as in vivo validation and application need to be carefully explored.

### 2.2. Anticancer

Cancer is a group of diseases characterized by the uncontrolled proliferation of abnormal cells in the body with the potential to spread to or invade other parts of the body. Although many different anticancer chemotherapies are already in clinical use, cancer is still a major disease burden in public healthcare and poses as the second leading cause of death worldwide [14]. While numerous anticancer strategies have been developed in recent years, natural products such as ginkgolic acids are alternatively merged as promising candidates for this demand.

In animal experiments, treatment with ginkgolic acid has shown a multi-aspects anticancer activity. The administration of ginkgolic acid has been proven to markedly reduce cancer cell proliferation in a dose- and time-dependent manner, arresting cell cycle at the G0/G1 phase of cell cycle, to inhibit cell migration and to induce apoptosis by activating caspase-9/-3 by upregulating the pro-apoptotic Bax protein and downregulating the anti-apoptotic Bcl-2 protein in various human cancer cell lines, including the tongue squamous carcinoma Tac8113 cell line, the human nasopharyngeal carcinoma 5-8F, the CNE2 and NP69 cell lines, as well as the human gastric cancer cell lines BGC-823, SGC-7901, MGC-803, and AGS [15,16,17]. Ginkgolic acid also exhibited, in a dose-dependent manner, the significant inhibition of invasion in human nasopharyngeal carcinoma cells of high aggressive and metastatic potentiality. For both human tongue and nasopharyngeal cancer cells, ginkgolic acid C15:1 induced apoptosis and was regulated by inhibiting AKT/NF-κB signaling, whereas suppressing STAT3/JAK2 signaling contributed to promoted apoptosis in human gastric cancer cells treated with ginkgolic acid C17:1 (20–80 µM) and in human multiple myeloma cells treated ginkgolic acid C17:1 (30–50 µM) [15,16,17,18]. These results suggest that the inductive activity of ginkgolic acid is associated with multiple cascades of signal transduction. Interestingly, in in vivo experiments in mice with gastric cancer xenografts, the intraperitoneal injection of ginkgolic acid C17:1 (7.5, 15, or 30 mg/kg) for 28 days significantly reduced gastric tumor growth in a dose-dependent manner, without significant body weight loss [16]. In the xenograft mouse model of nasopharyngeal carcinoma, the administration of ginkgolic acid C15:1 (15 mg/kg, 3 times/week) via gastric gavage for 3 weeks also exhibited an inhibitory effect on tumor growth. The co-administration of ginkgolic acid with 5-FU (5 mg/kg) resulted in a more profound inhibition of tumor growth than ginkgolic acid C15:1 or 5-FU alone, indicating the synergistic inhibition of nasopharyngeal cancer [17]. Similarly, cell proliferation and migration of ovarian cancer cells were remarkedly suppressed by ginkgolic acid C15:1 (≤20 ng/mL). Such anticancer effect was confirmed by inhibiting in vivo tumor growth after the administration of ginkgolic acid for 30 days. Both in vitro and in vivo results demonstrate that ginkgolic acid suppressed ovarian cancer by downregulating the lncRNA MALAT1/JAK2 axis [19]. Moreover, while ginkgolic acid C15:1 (1–100 µM) suppressed the viability of human pancreatic cancer cell lines Panc-1 and BxPC-3 as well as human hepatoblastoma HepG2 cells, or ginkgolic acid C17:1 (50 µM) inhibited the proliferation of human multiple myeloma U266 cells, they did not exhibit a measurable cytotoxic effect on non-cancer cells, i.e., normal human liver cell line HL-7702, human umbilical vein endothelial cells, or human peripheral blood mononuclear cells [18,20], although ginkgolic acid C15:1, at 20µM, impaired colony formation, migration, and invasion activities, as well as inhibited lipogenesis of Panc-1, BxPC-3, and HepG2 cells by activating AMPK signaling. In mouse model of xenograft pancreatic cancer, the administration of ginkgolic acid C15:1 (50 mg/kg, daily) via gastric gavage for 4 weeks showed dramatically reduced lipogenesis and remarkedly restrained tumor growth [20]. Similar anti-tumor effects were obtained in SW480 human colon cancer cells, where ginkgolic acid C15:1 (10–20 µmol/L) inhibited cell proliferation, migration, and invasion by inducing AMPK activity [21]. C17:1, another form of ginkgolic acid, was found to act as an effective suppressor of EGFR to sufficiently suppress tumor growth and invasion in human renal cancer 786-O and A498 cells (10–20 µM in vitro or 10 mg/kg twice per week for 6 weeks) and in human hepatoblastoma HepG2 cells (20–40 µg/mL in vitro or 40–80 mg/kg every other day for 2 weeks), by downregulating the EGFR/Akt/Erk signaling pathway [22,23]. Intriguingly, among the three major types of ginkgolic acids tested, ginkgolic acid C17:1 (IC_50_ = 8.5 µg/mL) demonstrated the strongest inhibition on cell viability and migration in human hepatocellular cancer SMMC7721 cells by inducing cell apoptosis through the upregulation of Bax expression and the activation of caspase-3 [24].

The abovementioned anticancer effects have been verified to link to SUMOylation with SUMO family members. Small Ubiquitin-like Modifier (SUMO) proteins are a conserved family of five proteins, from SUMO1 to SUMO5. In vertebrates, it consists of three ubiquitously expressed paralogs: SUMO1, SUMO2, and SUMO3. SUMOylation is a post-translational modification pathway by which SUMO is reversibly and covalently conjugated to selected proteins to regulate their properties and biological functions. It plays important roles in multiple biological processes, including DNA replication, DNA repair, transcriptional regulation, chromatin organization, cell cycle regulation, protein–protein interactions, protein–DNA interactions, and quality control of newly synthesized proteins. The clinical relevance of the SUMOylation pathway in human diseases has been firmly established and aberrant SUMOylation is closely related to the pathogenesis of various proliferative diseases such as cancer [25]. Notably, ginkgolic acids were found to be a potent inhibitor of protein SUMOylation by selectively blocking the formation of the E1-SUMO intermediate [26], and have been widely used as SUMO1-specific inhibitors in various studies in vitro and in vivo. Hamdoun and Efferth reported that 25 µM of ginkgolic acid C13:0 inhibited the migration in breast cancer cells through a mechanism of inhibiting the SUMOylation of NEMO (a key upstream regulator of NF-κB) and NF-κB activity [27]. Very recently, Meng and co-workers found a significant upregulation of SUMOylation in the tissues of uveal melanoma patients in comparison to the distal normal uvea tissues. The inhibition of SUMOylation by ginkgolic acid C15:1 is sufficient to induce apoptosis and reduce tumor growth both in vitro (dose of 50 µM) and in vivo (dose of 50 mg/kg via oral gavage) [28]. Another study on human tongue squamous carcinoma Tac8113 and Cal-27 cell lines revealed that ginkgolic acid C15:1 (5–10 µM) induced apoptosis activation and invasion repression through a mechanism of attenuating TGF-β1-triggered SMAD4 SUMOylation. In vivo experiments demonstrated that the administration of ginkgolic acid C15:1 (50 mg/kg, daily) via gastric gavage for 4 weeks remarkedly suppressed tumor growth [29]. In human cervical carcinoma-derived HeLa cells, ginkgolic acid C15:1 (20–50µM) significantly decreased the cell viability, induced mitochondrial fragmentation, and promoted mitophagy (selective autophagy), speculatively through the inhibition of SUMOylation [30]. Using breast cancer cell lines (MCF7, MDA-MB-231, and BT474), human prostate cancer LnCap and 22Rv1 cell lines, and in vivo breast cancer xerographs, Lorente and co-workers verified that SUMO1 inhibition by ginkgolic acid C15:1 (10µM in vitro or 10 mg/kg daily for 18 days) dampened the tumorigenic properties of cancer cells through two different mechanisms simultaneously: stimulating autophagy-mediated cancer cell death and inhibiting tumor invasion via the regulation of RAC1 SUMOylation [31]. Hence, the inhibitory effects of SUMOylation by ginkgolic acid may play a fundamental role in anticancer activity.

The phosphoinositide 3-kinase (PI3K)/Akt/mechanistic target of the rapamycin (mTOR) signaling pathway is highly associated with cell growth, proliferation, survival, autophagy, and metabolism [32]. The PI3K/Akt /mTOR signaling pathway negatively regulates the autophagic process and is frequently hyperactivated with close correlation to survival and metastasis in human cancers [33,34]. In lung cancer cell lines A549 and H1299, ginkgolic acid C15:1 at 100 µM suppressed cell viability and repressed invasion and migration through the mechanism of inactivating the PI3K/Akt /mTOR signaling pathway [35]. Similar cancer suppressive effects have been observed in colon cancer SW480 cells and in in vivo xerographs. Ginkgolic acid C15:1 (12–64 µM) diminished cancer cell migration and invasion, and induced cell death by activating caspase-3 in apoptosis and arresting cells at the G0/G1 phase. Notably, the significant reduction in p-mTOR indicated a sequential increased autophagy upon ginkgolic acid treatment. Experiments in colon cancer cells have verified that the blockage of autophagosome formation enhanced apoptosis induced by ginkgolic acid, indicating that ginkgolic acid C15:1 impacted apoptosis more than autophagy. Further investigation revealed that ginkgolic acid triggered interplay between apoptosis and autophagy, which was mediated by ROS generation [36]. Likewise, the inhibition of the PI3K/Akt/mTOR pathway by ginkgolic acid C15:1 (20–100 µM in vitro or 25 mg/mL intraperitoneal injection for 18 days) led to the activating of apoptosis and autophagy of endometrial carcinoma cells both in vivo and in vitro [37]. In human hepatoblastoma HepG2 cells, ginkgolic acid C17:1 (20–80 µg/mL in vitro, or 20–80 mg/kg every other day intraperitoneally for 2 weeks) dose-dependently inhibited the cell viability, migration, and invasion, as well as tumor growth in vivo [38]. Inhibiting the activation of the MAPK/MMP, Rho/Rho-associated protein kinase and PI3K/Akt/mTOR signaling pathways correlates to ginkgolic acid C17:1-induced tumor suppression. Therefore, these findings underscore the important negative engagement of the PI3K/Akt/mTOR signaling pathways in the ginkgolic acid-induced apoptosis and cell migration, and thus, anticancer activity.

Cisplatin is one of the commonly used anticancer drugs in clinic chemotherapy, but drug resistance is a major therapeutic obstacle for successful treatment. While ginkgolic C17:1 alone induced autophagy and apoptosis in the human hepatoblastoma cells HepG2, it was found to paradoxically inhibit cisplatin-induced autophagy by inhibiting the AMPK/ULK1 pathway. Here, it is interesting to note that the inhibition of PI3K/Akt/mTOR signaling by ginkgolic acid C17:1 stimulated cisplatin-induced apoptosis rather than autophagy in HepG2 cells [39]. This finding may be explained by the crosstalk between autophagy and apoptosis because they share numerous common regulatory molecules, including Bcl-2 and Beclin-1, and the PI3K/Akt/mTOR signaling pathway [40,41,42]. Moreover, further studies comparing hepatoblastoma cells with normal hepatocytes showed that ginkgolic acid C17:1 monotreatment at a concentration lower than 80 µg/mL significantly inhibited the viability of hepatoblastoma cells HepG2 but had no cytotoxicity in normal hepatocytes L02. In comparison with cisplatin alone, the combination of ginkgolic acid C17:1 (<80µg/mL) with a low dose of cisplatin (2 µg/mL) exhibited a profound cytotoxicity enhancement in hepatoblastoma cells, but reversed cisplatin-induced apoptosis to almost no toxicity in normal hepatocytes. Importantly, in normal hepatocytes, autophagy was activated by ginkgolic acid C17:1 alone or in conjugation with cisplatin; however, despite ginkgolic C17:1 alone inducing autophagy in liver cancer HepG2 cells, it did remarkably inhibit the cisplatin-induced autophagy through the inhibition of the AMPK/ULK1 pathway. Ginkgolic C17:1 or cisplatin alone inhibited the PI3K/AKT/mTOR pathway in hepatoblastoma HepG2 cells, and co-treatment exhibited an enhanced inhibitory effect, while in normal hepatocytes, ginkgolic C17:1 alone did not significantly impact this signaling pathway, rather reducing cisplatin-caused inhibition to prevent cell damage [43]. Such differential effects in hepatoblastoma cells and normal hepatocytes propose ginkgolic C17:1 as a promising candidate for combined chemotherapy.

The multi-aspects actions and underlying mechanisms for anticancer activities by ginkgolic acids are summarized in Table 1.

### 2.3. Antimicrobials, Anti-Virus, and Anti-Inflammation

Since thousands of years, *Ginkgo biloba* has been used in traditional medicine for treating skin infection, bronchitis, asthma, etc. In the last decade, studies have shown that ginkgolic acids also exhibit antimicrobial, anti-inflammatory, and anti-viral activities.

Biofilm formation is not only closely related to bacterial infection but also to a mechanism for antimicrobial resistance. Studies on the most common Gram-positive bacteria *Staphylococcus aureus*, *Cutibacterium acnes* (formerly, *Propionibacterium acnes*), and *Streptococcus pyogenes* revealed that ginkgolic acid C15:1 at 16 μg/mL could inhibit the biofilm formation and growth of *Cutibacterium acnes*, whereas a concentration lower than 16 μg/mL of ginkgolic acid C15:1 could inhibit the growth of all these bacteria species [44].

*Escherichia coli* O157:H7, a Gram-negative bacterium and major foodborne pathogen, causes severe human diseases that presently lack effective therapy. Lee and co-workers found that ginkgolic acid C17:1 at 5 μg/mL significantly inhibited the fimbriae production and biofilm formation of *Escherichia coli* O157:H7 with no effect on bacteria growth [45]. Another Gram-negative bacterium, *Pseudomonas aeruginosa*, is a predominantly antibiotic-resistant pathogen for life-threatening infections in the human respiratory system, burns and wounds, the urinary tract, as well as medical implant devices [46]. Recent studies showed that ginkgolic acids could reduce the pyocyanin production of *Pseudomonas aeruginosa* in a dose-dependent manner. Ginkgolic acid C17:1 at the concentration of 100 µg/mL inhibited approximately 90% of pyocyanin production. Such inhibitory activity on pyocyanin virulence was also observed in human lung epithelial A549 cells and nematode *C. elegans* as model host, with no or slight cytotoxicity. Moreover, ginkgolic acids induced *Pseudomonas aeruginosa* membrane stiffness [47].

**Figure 3:**
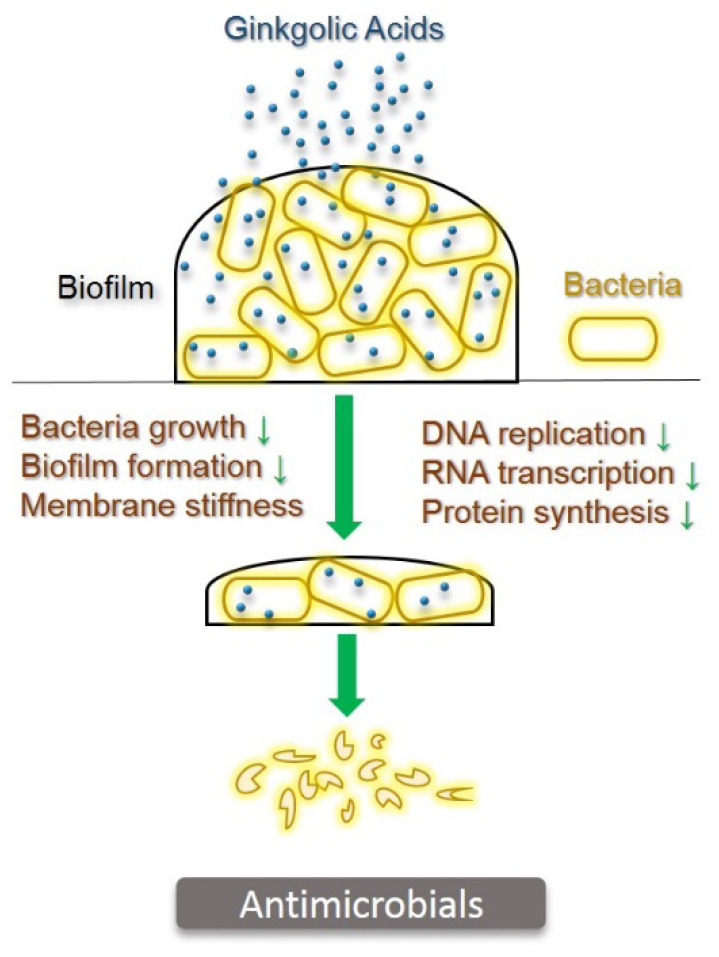
Antimicrobial activity of ginkgolic acids. Ginkgolic acids penetrate through biofilm and bacteria cell walls into cytoplasm to kill bacteria via inhibition of growth, biofilm formation, DNA replication, RNA transcription, protein synthesis, and induction of membrane stiffness.

Investigations on the underlying mechanisms of antibacterial activity indicated that a large amount of ginkgolic acid C15:1 could, within a very short time, penetrate Gram-positive bacteria to inhibit DNA replication, RNA transcription, and protein synthesis [48]. The reason for the little antibacterial activity in Gram-negative bacteria could be due to the interception of the majority of the ginkgolic acid C15:1 by lipid-soluble components (including lipopolysaccharides and phospholipids) in the cell wall. The difference of chemical structures may warrant ginkgolic acid C17:1, penetrable and poise, to enter the cytoplasmic compartment through the cell wall, thus exhibiting antibacterial activity in Gram-negative bacteria (Figure 3).

**Figure 4:**
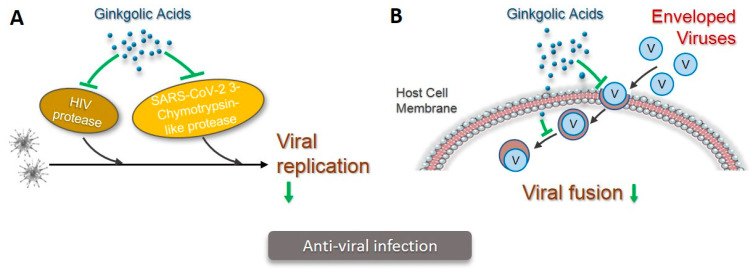
Mechanisms for anti-viral infection of ginkgolic acids. A. Ginkgolic acids inhibit the activity of HIV protease or SARS-CoV-2 3-Chymotrypsin-like protease, leading to inhibition of viral replication of HIV or multiple coronaviruses (illustrated in green). B. Ginkgolic acids inhibit viral fusion of a broad spectrum of enveloped viruses to host cell either on cell surface or intracellular compartments (illustrated in green).

Human immunodeficiency virus-1 (HIV-1) protease plays a crucial role in the life cycle of HIV, as it cuts up large precursor proteins into smaller proteins for HIV replication. In a cell-free system, ginkgolic acid effectively inhibited HIV protease activity in a concentration-dependent manner and in a range from 31.2 to 125 µg/mL. In addition, ginkgolic acid at 50 and 100 μg/mL showed a concentration-dependent inhibition of the HIV infection in human peripheral blood mononuclear cells (PBMCs), without any cytotoxicity to the host cells [49]—another one of the important processes for the life span of enveloped viruses is viral fusion. Enveloped viruses infect host cells by a membrane fusion reaction that takes place at the cell surface or in intracellular compartments following virus uptake. A total of 10µM ginkgolic acid C15:1 inhibited the viral fusion of human cytomegalovirus (HCMV), both the viral fusion and viral protein synthesis of the herpes simplex virus type 1 (HSV-1) and the Zika virus (ZIKV), as well as the entry of a non-enveloped human adenovirus. Moreover, ginkgolic acids C13:0, C15:1, and C17:1 at 10 µM individually completed the blocked viral fusion of a broad spectrum of enveloped viruses, including the Zika virus (ZIKV), the human immune deficiency virus (HIV), the Ebola virus (EBOV), the influenza A virus (IAV), the Semliki forest virus (SFV) E1/E2, the Venezuelan equine encephalitis virus (VEEV), the vesicular stomatitis virus (VSV) G, and the Epstein–Barr virus (EBV) [50]. Most interestingly, recent studies indicated an effective inhibitory effect by ginkgolic acid C17:1, with IC50 values of less than 2 μM, on SARS-CoV-2 3-Chymotrypsin-like protease, a main proteinase that plays essential role in the viral replication of a broad spectrum of coronaviruses [51]. This finding adds a new type of protease as the target for the anti-viral activity of ginkgolic acids (Figure 4).

**Figure 5:**
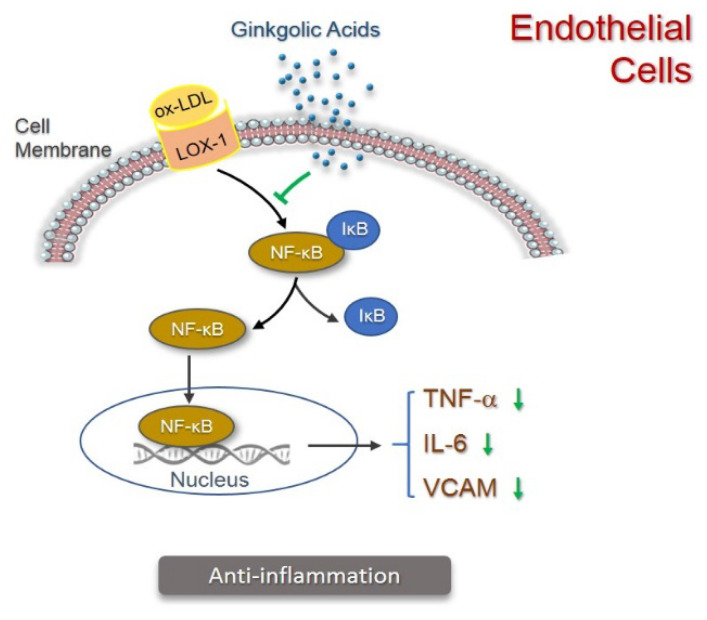
Anti-inflammation activity of ginkgolic acids in endothelial cells. Binding of oxidized low-density lipoprotein (oxLDL) to its transmembrane protein Lox-1 triggers release of NF-κB from its inhibitor IκB and migration of NF-κB to nucleus, thus enhancing transcription of TNF-α, IL-6, and VCAM-1 (illustrated in black). Ginkgolic acids inhibit NF-κB signaling, thereby inhibiting the production of TNF-α, IL-6, and VCAM-1 (illustrated in green).

Ginkgolic acid C15:1 has also been reported to protect human cells against oxidized low-density lipoprotein-induced inflammation (Figure 5). In human microvascular endothelial cells, ginkgolic acid up to 50µM significantly inhibited the expression of TNF-α (tumor necrosis factor-α), IL-6 (interleukin-6), and VCAM-1 (vascular cell adhesion molecular-1) induced by oxidized low-density lipoproteins, in a dose-dependent manner. Similar results were obtained in human peripheral blood mononuclear cells treated with 5µg/mL and 50µg/mL of ginkgolic acid. This anti-inflammatory effect in both human cell lines and primary blood monocytes was shown to be mediated by the inhibited NF-κB signaling pathway [52].

### 2.4. Anti-Fibrosis

Heart failure is a major public health issue with increasing prevalence worldwide. Myocardial infarction (MI) caused by a lack of blood flow to the heart muscle due to coronary artery occlusion is the leading cause of heart failure as well as life-threatening. Myocardial fibrotic remodeling occurs as a mechanism in response to MI, but consequently over-proliferated fibroblasts/myofibroblasts and abnormally deposited collagen impair heart function. In a mouse model of MI, the administration of ginkgolic acid (C17:1, 50 mg/kg/d) for 3 days before MI suppressed collagen production and fibroblasts activation. Moreover, the pre-treatment of ginkgolic acid significantly improved heart function in terms of ejection fraction and fractional shortening. Notably, the enhanced SUMOylation in this MI mouse model was inhibited by the pre-treatment of ginkgolic acid, indicating that the prevention of myocadiac fibrosis may be related to the inhibition of SUMOylation by ginkgolic acid [53].

A similar anti-fibrotic effect of ginkgolic acid has been recently reported in the lungs. Idiopathic pulmonary fibrosis (IPF) is a chronic respiratory disease with unknown etiology but often progressive to fatal interstitial lung disease [54,55]. It is imperative to discover/develop new therapeutic regimens because there only exist two drugs approved for idiopathic pulmonary fibrosis and their efficacy is limited, with evident gastrointestinal side effects [56,57]. Similarly in the heart, the findings of significantly increased protein levels of SUMO1, SUMO2/3, and UBC9 in the lung tissue of IPF patients compared with those of healthy lung tissue link pulmonary fibrosis to SUMOylation. In a mouse model of bleomycin-induced pulmonary fibrosis, ginkgolic acid (25 mg/kg/d) was administered 24 h after the induction of pulmonary fibrosis, and a significant reduction in pulmonary fibrosis was observed in the ginkgolic acid-treated group, which is suggestive of the in vivo anti-fibrosis effect of ginkgolic acid. Furthermore, in vitro experiments in lung tissue and lung epithelial A549 cells revealed that ginkgolic acid inhibited the TGF-b1-mediated SUMOylation of SMAD4 and epithelial–mesenchymal transition, thus achieving protective effects against pulmonary fibrosis [58]. Both in vivo and in vitro studies suggest an anti-fibrotic potential of ginkgolic acid by augmenting SUMOylation as well as therapeutic perspectives in the future of anti-fibrosis clinical testing.

### 2.5. Cardiovascular Protection

Hypertension is a major healthcare problem globally. It often leads to cardiovascular disease complications, including myocardial infarction, heart failure, and stroke, and other end-organ diseases such as chronic kidney disease and renal failure. Endothelial dysfunction and reduced nitric oxide (NO) bioactivity represent prominent pathophysiological abnormalities associated with hypertensive cardiovascular disease [59]. It has been reported that 30µM ginkgolic acid C15:1 significantly increased NO production in human umbilical vein endothelial cells, and with 60µM remarkedly improved vasodilatation of aorta from Ang II-induced hypertensive mice [60]. These in vitro data indicated that ginkgolic acid may have a protective effect against endothelial dysfunction, but its clinical application needs to be further verified.

### 2.6. Renoprotection

Chronic kidney disease is a multifactorial disease mainly caused by diabetes and hypertension. The prevalence of chronic kidney disease has greatly increased in the last two decades and has a significant impact on worldwide healthcare. An important etiology of chronic kidney disease is IgA nephropathy, the most common form of primary glomerulonephritis characterized by mesangial cell proliferation and mesangial matrix expansion [61]. Studies have shown that many factors including autophagy are involved in the pathogenesis of IgA nephropathy [62,63]. In rats with IgA nephropathy, mesangial cell proliferation and renal lesions are linked to the reduced autophagy that is mediated by the mTOR-related signaling pathway. Thus, impaired or deficient autophagy is considered to be causally related to kidney disease.

In recent years, the alteration of the SUMOylation process has been found to be linked to kidney diseases. For instance, a decrease in SUMOylation protects renal tubular cells in acute kidney injury [64] and relates to normal localization, stability, and the proper function of nephrin [65]. Very recently, Tan et al. found upregulated SUMO1 expression in biopsy tissues from IgA nephropathy patients in comparison with the minor glomerular abnormality control group, and a high SUMO1 expression in the mesangial area of the mouse model of IgA nephropathy [66]. In a cell model of IgA nephropathy, the inhibition of SUMOylation by 2 µmol/L ginkgolic acid C15:1 could reduce SUMO1 expression, promote autophagy, and lessen cell proliferation of human mesangial cells, indicating a reno-protective effect of ginkgolic acid in IgA nephropathy. Although this is the first report on renoprotection by ginkgolic acid, it would be interesting in the future to translate this result into animal models of kidney disease.

### 2.7. Neuroprotection

Neurodegenerative disease-specific proteins are often SUMOylated within pathological protein aggregates in the brain [67]. SUMOylation is linked to the pathology of neurodegenerative diseases, including Alzheimer’s disease, Huntington’s disease, and Parkinson’s disease [68,69,70,71,72,73].

Alzheimer’s disease (AD), the most common form of dementia in the elderly, is characterized by the deposition of insoluble protein aggregates of amyloid-β (Aβ) and the microtubule-associated protein tau in the brain, accompanied by the progressive loss of neurons. Before the formation of amyloid plaques and neuron loss, early synaptic impairment already occurs in different brain areas, including those that are involved in cognitive process and memory formation [74]. Ginkgolic acid C15:1 at concentrations from 3 to 30 µM could significantly increase the long-term potentiation (a molecular mechanism which underlies learning and memory) in mouse hippocampal CA1 pyramidal neurons. Furthermore, the co-application of 1 µM of ginkgolic acid and 200 nM of Aβ revealed that ginkgolic acid rescued Aβ-induced long-term potentiation impairment [75]. As SUMOylation is among the mechanisms for Aβ-induced synaptic dysfunction [76,77], the inhibition of SUMOylation may contribute to the neuroprotective effects of ginkgolic acid against Aβ-induced impairment of neurotransmitter release and synaptic plasticity.

The neuropathological hallmark of Parkinson’s disease (PD) is the widespread intracellular inclusion bodies (Lewy bodies) and neurotic deposits (Lewy neurites) of phosphorylated α-synuclein. Results from immunoprecipitation revealed strikingly increased levels of SUMOylated α-synuclein in the cerebral cortex of Parkinson’s disease patients with dementia [78]. Interestingly, in potassium chloride-induced α-synuclein aggregates in SH-SY5Y neuroblastoma cells, while 10 µM ginkgolic acid C15:1 upregulated autophagy, it had little effect on SUMOylation. These results indicate that the clearance of pre-formed intracellular α-synuclein aggregates by ginkgolic acid C15:1 is autophagy-dependent [79]. Other mechanisms than SUMOylation may be involved in the ginkgolic acid-induced clearance of α-synuclein.

Huntington´s disease (HD), an inherited neurodegenerative disorder, is caused by the expansion mutation of the CAG repeat in the HTT gene, which leads to the translation into the mutant huntingtin protein (mHTT), which confers a predominant toxic gain-of-function mechanism to degenerate nerve cells. As the SUMOylation of mHTT makes it more soluble and more toxic, a novel approach to therapy may be the attempt to clear the mHTT protein from nerve cells. In the comparing studies on mouse striatal neuron cells (normal and HD type) and human fibroblasts from healthy or HD patients, ginkgolic acid C15:1 treatment at 100 μM remarkedly increased autophagy activities in both the normal and HD cell types tested. Meanwhile, ginkgolic acid exhibited a strong diminishment of mHTT in HD striatal neuron cells, and this diminishing effect was even more pronounced in the human HD fibroblasts. This clearance effect is mHTT protein-specific because either normal HTT protein or mTOR levels were not affected by ginkgolic acid in WT striatal neuronal cells or human healthy fibroblasts [80]. These results suggest that ginkgolic acid-mediated SUMOylation inhibition enhances autophagy activities and diminishes the mHTT protein in both mouse neuronal HD cells and human HD fibroblasts. Taken together, such neuroprotective effects of ginkgolic acids suggest a promising therapeutic potential of ginkgolic acid on neurodegenerative diseases.

## 3. Autophagy in Pharmacological Activities of Ginkgolic Acids

Autophagy is a revolutionary highly conserved cellular process that plays a crucial role in maintaining homeostasis in cells. In normal cells, autophagy, through its general (non-selective) and selective mechanisms, degrades damaged organelles, toxic unfolded proteins, and oncogenic protein substrates to keep “quality control”. In addition, it also serves as a cellular adaptive response to manage stressful conditions such as starvation, stress, and infection [81]. Depending on the specific cellular context, stimulating autophagy can be either protective or cytotoxic to a cell. The dysregulation of autophagy is closely related to diverse diseases such as neurodegeneration, cancer, heart dysfunction, and infectious diseases [82,83]. The importance of autophagy in health and disease was strongly highlighted in 2016 when Dr. Yoshinori Ohsumi was awarded the Nobel Prize for Physiology or Medicine in recognition for his work on elucidating the mechanism of autophagy and its impact on the study of human health and disease [84].

Autophagy is of importance in tumorigenesis and progression. It is thought that autophagy stimulation may be beneficial to prevent normal cells from cancer, but once cancer has developed, autophagy may facilitate cancer cell survival [81]. The role of autophagy in the pathogenesis of cancer is often regarded as a “double-edged sword” in the initiation, development, and metastasis of cancer, depending on its stages [85,86,87]. Autophagy and apoptosis are two important programmed cell death processes in cancer cells. Apoptosis, characterized by plasma membrane blebbing, cell shrinkage, chromatin condensation, and the formation of apoptotic bodies, as well as caspases activation, is a significant cellular mechanism that ultimately contributes to chemotherapy-induced cancer cell death [88]. A complex signaling network is often formed by the interactions among the components of apoptosis and autophagy upon similar stimuli [89,90]. Under specific biological conditions, the crosstalk between autophagy and apoptosis can lead to a collaboration for cellular demise. The key regulatory molecules shared between these two processes include Bcl-2 and Beclin-1, as well as the PI3K/Akt/mTOR signaling pathway [40,41,42]. Beclin-1 (a putative tumor suppressor), the microtubule-associated protein 1 light chain 3 (LC3), and the adaptor sequestosome 1 (SQSTM1; p62) are commonly used biomarkers of autophagy. An increase in Beclin-1 or LC3 usually represents autophagy activation, whereas p62 increases upon autophagy inhibition [85,86,91].

Autophagy is regulated by different pathways, among which the PI3K/Akt/mTOR signaling pathway and the AMPK/ULK1 signaling pathway are two key regulators [32,92]. Under conditions such as energy deficiency and hypoxia, the PI3K/Akt/mTOR signaling pathway negatively regulates autophagy by mediating mTOR expression. The serine/threonine protein kinase AMPK can positively regulate autophagy by phosphorylating ULK1 at multiple and specific sites. The conjugation of mTOR to ULK1 inhibits ULK1 activity, which in turn suppresses the interaction between ULK1 and AMPK, thus inhibiting the AMPK/ULK1 signaling pathway. On the other hand, AMPK phosphorylates the tuberous sclerosis complex-2 tumor suppressor to inhibit the mTOR pathway [93].

**Figure 6:**
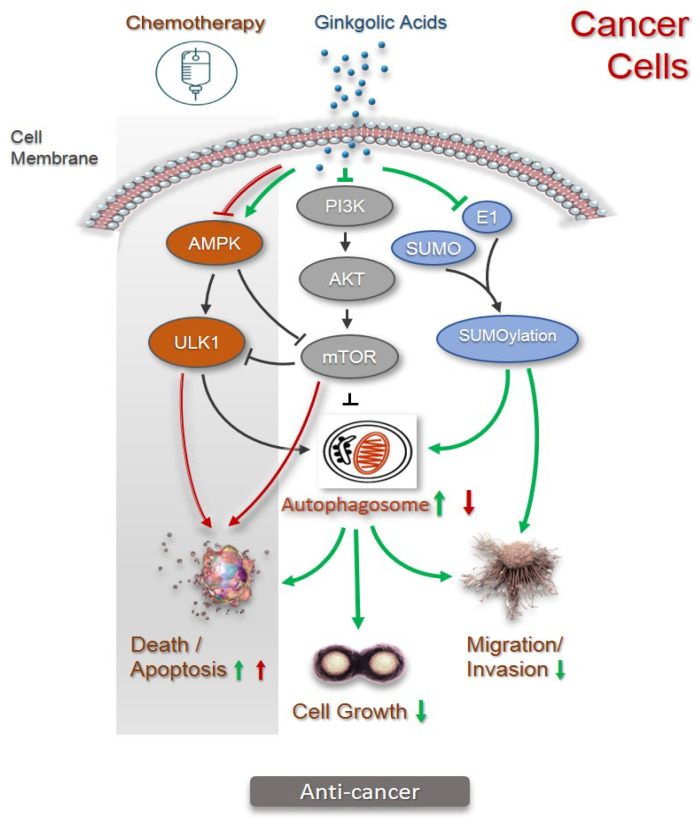
Role of autophagy in mechanisms for pharmacological activities of ginkgolic acids in cancer cells. As the key regulators, the PI3K/Akt/mTOR signaling pathway negatively and the AMPK/ULK1 signaling pathway positively regulate autophagy (illustrated in black). Through inhibition of PI3K/Akt/mTOR pathway and activation of AMPK/ULK1 pathway, ginkgolic acids upregulate autophagy (illustrated in green). SUMOylation is activated by SUMO-activating enzyme (E1)-initiated cascade that adds SUMO peptides to targeted proteins (illustrated in black). Functioning as SUMO1-specific inhibitor, ginkgolic acids inhibit SUMOylation, thus suppressing migration and invasion of cancer cells. Activation of autophagy by ginkgolic acids inhibits cancer cell growth, migration, and invasion and promotes cancer cell death (illustrated in green). When co-administered with chemotherapy, ginkgolic acids sensitize the cancer cells to chemotherapy by suppressing the chemotherapy-induced autophagy by inhibiting the AMPK/ULK1 pathway and/or stimulating chemotherapy-induced apoptosis via inhibition of PI3K/Akt/mTOR pathway (illustrated in red).

Growing evidence shows that autophagy is impaired in many tumor types; therefore, the loss of autophagy is a hallmark of cancer and autophagy is considered as a tumor suppressor in cancer [87,94]. Through different mechanisms, ginkgolic acids induce cancer cell death and inhibit tumor growth in a variety of cancers from tongue, nasopharynx, uveal melanoma, lung, liver, pancreas, kidney, prostate, colon, multiple myeloma, breast, ovarian, gastric, endometrial, and cervical carcinoma (Table 1). Meanwhile, the ginkgolic acid-induced activation of autophagy has been verified by autophagy biomarkers in lung, colon, liver, endometrial carcinoma cells, as well as cervical carcinoma-derived HeLa cells, and autophagy-mediated cancer cell death has been confirmed in breast cancer upon ginkgolic acid treatment. The inhibition of protein SUMOylation was seen in ginkgolic acids-treated tongue, uveal melanoma, and breast cancers. Since SUMO1 inhibition is known to promote autophagy activation [31,80], ginkgolic acid, as a SUMO1 specific inhibitor, is capable of activating autophagy by suppressing SUMOylation. The currently available data suggest the activation of autophagy by ginkgolic acids in cancer cells and a likely cooperation with apoptosis for cancer cell death, thus suppressing cancer cell growth, migration, and invasion (Figure 6). In line with ginkgolic acids, other natural compounds such as daphnetin also induce apoptosis and activate autophagy in cancer cells [95].

The role of autophagy in cancer is complicated and often competing. On one hand, autophagy activation suppresses cancer, and on the other hand, chemotherapy-induced autophagy leads to cancer drug resistance, which is an obstacle for the long-term effectiveness of chemotherapy [96,97]. Five-fluorouracil (5-FU) is a chemotherapy drug that has been widely used to treat different types of cancers, but it has limited clinical applications due to drug resistance developed after treatment [98]. A combination of 5-FU with other therapeutic agents is known to increase the drug response in cancer [99]. The co-administration of ginkgolic acid with 5-FU leads to the synergistic suppression on nasopharyngeal cancer that is evidenced by a more profound inhibition of tumor growth than ginkgolic acid or 5-FU alone [17]. Cisplatin is another commonly used chemotherapy with the same problem of drug resistance which may occur during long-term treatment. In hepatoblastoma cells, while cisplatin alone induces autophagy, the co-treatment of ginkgolic acid with cisplatin inhibits cisplatin-induced autophagy and enhances cisplatin-induced apoptosis. The inhibition of cisplatin-induced autophagy increases cisplatin-induced apoptosis, suggesting the synergistic induction on cancer cell death via inhibiting cisplatin-induced autophagy activity [39,43]. Thus, through the inhibition of chemotherapy-induced autophagy, ginkgolic acids can enhance the sensitivity of cancer cells to chemotherapy. In perfect agreement, 3-methyladenine (3-MA), an inhibitor of autophagy, when used in combination with cisplatin, enhances cisplatin-induced apoptosis in the MG63 human osteosarcoma cell line [42].

It has been shown that autophagy plays a critical role in the homeostasis of lung epithelia [100]. The finding that the restoration of Beclin-1 expression rescues the cystic fibrosis phenotype in mice suggests the pathogenic involvement of impaired autophagy in lung disease. Ginkgolic acids can reduce pulmonary fibrosis both in vitro and in mouse models through the inhibition of the TGF-b1-mediated SUMOylation of SMAD4 and epithelial–mesenchymal transition [58]. As SUMOylation inhibition is closely associated with autophagy activation [31,80], although autophagy was not investigated in this study, it is more likely that autophagy is involved in the anti-fibrotic effect of ginkgolic acids.

Autophagy is required to maintain the integrity of podocytes and tubular cells in the kidney. Impaired or deficient autophagy may contribute to kidney diseases such as glomerulosclerosis [85]. Ginkgolic acids exhibit protective effects on IgA nephropathy, a chronic glomerulosclerosis, by inhibiting SUMOylation that leads to autophagy activation and lessening the proliferation of mesangial cells [66].

Aggregate forms of a-synuclein in Parkison´s disease, aggregates of amyloid-β (Aβ) in Alzheimer’s disease, and expanded polyglutamine (polyQ)-containing protein (mHTT) in Huntington´s disease are well-known hallmarks of the corresponding neurodegenerative diseases. The accumulation of autophagic vacuoles in human neurodegenerative diseases provides evidence that the clearance of such misfolded, aggregate proteins is highly dependent on autophagy [101,102]. In animal models, such as for Huntington disease, the pharmacologic upregulation of autophagy effectively reduces neuronal aggregates and suppresses the progression of neurological symptoms [103]. Recently, natural ginkgolic acid products exhibit promising neuroprotection activity by the effective clearance of neuronal aggregates by upregulating autophagy that is mainly mediated by inhibiting SUMOylation [32,75].

**Figure 7:**
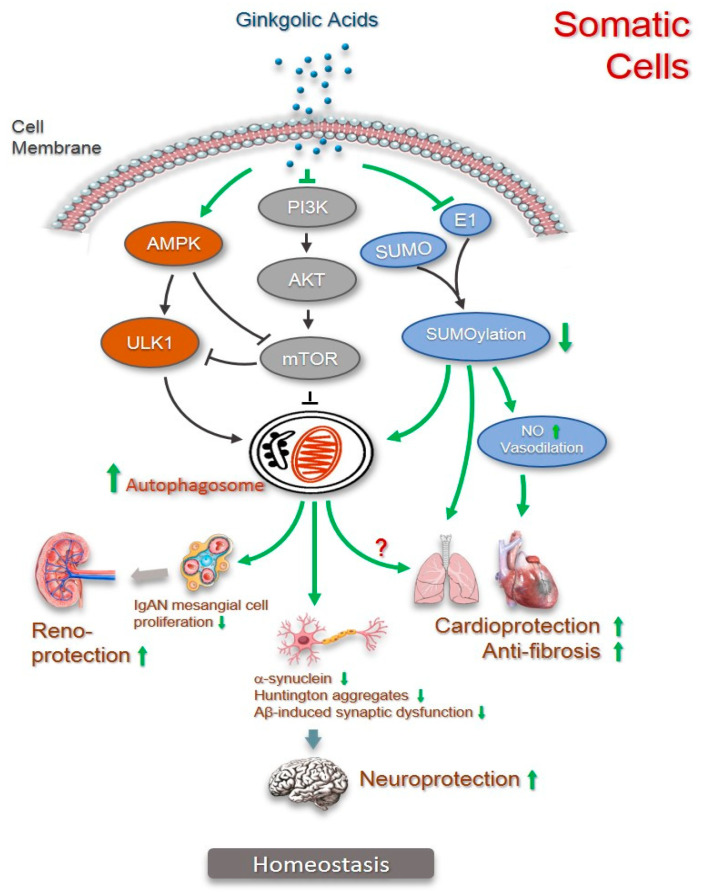
Involvement of autophagy in the mechanisms for anti-fibrosis, cardioprotection, renoprotection, and neuroprotection by ginkgolic acids. As the key regulators, the PI3K/Akt/mTOR signaling pathway negatively and the AMPK/ULK1 signaling pathway positively regulate autophagy (illustrated in black). Through inhibition of PI3K/Akt/mTOR pathway and activation of AMPK/ULK1 pathway, ginkgolic acids upregulate autophagy (illustrated in green). SUMOylation is activated by SUMO-activating enzyme (E1)-initiated cascade that adds SUMO peptides to targeted proteins (illustrated in black). Functioning as SUMO1 specific inhibitor, ginkgolic acids inhibit SOMUylation by inhibiting E1, leading to increase in nitric oxide (NO) and vasodilation improvement. Inhibition of SUMOylation also activates autophagy to prevent myocadiac fibrosis. Since SUMOylation inhibition contributes to the protection of lung fibrosis by ginkgolic acids, autophagy activation caused by SUMOylation inhibition cannot be excluded. Ginkgolic acids-induced SUMOylation inhibition leads to upregulation of autophagy in IgA nephropathy (IgAN) that lessens proliferation of mesangial cells (illustrated in green). The same mechanism applies to the clearance of α-synuclein and mutant Huntington protein aggregates as well as Aβ-induced synaptic dysfunction rescue in neuronal cells (illustrated in green).

The involvement of autophagy in anti-fibrosis, cardiovascular protection, renoprotection, and neuroprotection by ginkgolic acids is summarized in Figure 7.

## 4. Conclusions

Botanical natural ginkgolic acid products have shown a wide range of pharmacological activities, including antidiabetic, anticancer, anti-fibrosis, anti-bacteria, anti-virus, and reno/neuroprotective effects. To date, the available data support a dual role of autophagy in the pharmacologic activities of ginkgolic acids. In various cell types of different pathological conditions, including neurodegeneration and cancer, ginkgolic acids generally activate autophagy to reverse the condition toward normality or to suppress cancer. When co-applied with chemotherapy, ginkgolic acids inhibit chemotherapy-induced autophagy to sensitize cancer cells’ response to chemotherapy. In the cancer therapeutic area, it is generally considered that autophagy may facilitate cancer cell survival. Indeed, the inhibition of autophagy is already attempted in clinical trials for cancer therapy. However, the fact that ginkgolic acids activate autophagy to suppress cancer both in vitro and in vivo reflects the complex role of autophagy in cancer. Therefore, the challenge of manipulating autophagy as cancer therapy remains: should we inhibit or activate autophagy in cancer? On the other hand, excitingly, ginkgolic acids in combination with chemotherapy 5-FU or cisplatin exert the synergistic suppression of cancer and protect non-cancerous cells from chemotherapy. This means that adding ginkgolic acid could enhance the efficacy and/or relieve the side effects of chemotherapeutic drugs, thus bringing us the hope to improve the current treatment, particularly regarding drug resistance due to chemotherapy. Although the underlying mechanisms remain largely unknown and may vary in different pathological conditions, the major ginkgolic acids C13:0, C15:1, and C17:1 exhibit common anticancer activities as well as neuroprotective and antiviral activities, thus potentially representing clinically interesting candidates in novel drug development or combined chemotherapy in human diseases. 

## Figures and Tables

**Table 1:** Anticancer activities of major ginkgolic acids.

Ginkgolic Acids	Cancer Cell Types	Actions and Mechanisms	References
C15:1	Human tongue squamous Tac8113	Inhibit cell growth via reduction in the Bcl-2/Bax ratio and stimulation of caspase-3 activity; suppress tumor growth in mouse xenografts	Ref. [15]
C17:1	Human gastric BGC-823, SGC-7901, MGC-803 and AGS	Suppress cancer growth by inducing apoptosis and suppressing STAT3/JAK2 signaling regulated by ROS, both in vitro and in mouse xenografts	Ref. [16]
C15:1	Human nasopharyngeal 5-8F, CNE2 and NP69	Suppress cancer growth by inducing apoptosis through inhibition of AKT/NF-κB signaling, both in vitro and in mouse xenografts; synergistically suppress cancer with 5-FU	Ref. [17]
C17:1	Human multiple myeloma U266ata	Promote apoptosis via suppressing STAT3/JAK2 signaling	Ref. [18]
C15:1	Human ovary SKOV3 and CAOV3	Inhibit proliferation and migration through suppressing lncRNA MALAT1/JAK2 axis activity, and suppress tumor growth in mouse xenografts	Ref. [19]
C15:1	Human pancreas Panc-1 and BxPC-3	Suppress colony formation, migration, invasion, and lipogenesis through activating AMPK signaling; inhibit tumor growth in mouse xenografts	Ref. [20]
C15:1	Human colon SW480	Reduce proliferation, migration, and invasion through stimulating AMPK signaling	Ref. [21]
C17:1	Human liver hepatoblastoma HepG2	Reduce proliferation, migration, and invasion via inhibiting EGF-induced activation of PI3K/Akt signaling pathways; inhibit tumor growth in mouse xenografts	Ref. [22]
C17:1	Human kidney 786-O and A498	Suppress proliferation and invasion via inactivating epidermal growth factor receptor (EGFR) signaling pathway; inhibit tumor growth in mouse xenografts	Ref. [23]
C13:0, C15:1, C17:1	Human liver SMMC7721	Induce apoptosis via caspases-3 activity, upregulate Bax expression; inhibit migration	Ref. [24]
C13:0	Human breast MCF-7 and MDA-MB-231	Inhibit migration by inhibition of NEMO SUMOylation and NF-κB activity	Ref. [27]
C15:1	Human uveal melanoma OCM3, OMM2.3 and Mel285	Induce apoptosis through inhibition of SUMOylation; inhibit tumor growth in mouse xenografts	Ref. [28]
C15:1	Human tongue squamous Tac8113 and Cal-27	Induce apoptosis and suppress invasion through inhibition of TGF-β1-induced enhancement of SUMOylation of SMAD4; inhibit tumor growth in mouse xenografts	Ref. [29]
C15:1	Human cervical carcinoma derived HeLa	Impair mitochondrial function by decreasing mitochondrial biogenesis, promote mitophagy	Ref. [30]
C15:1	Human breast MCF7, MDA-MB-231 and BT474; human prostate LnCap and 22Rv1	In vitro and in vivo inhibition of SUMO1-mediated SUMOylation, induce autophagy-mediated cancer cell death and reduce invasion via RAC1; inhibit tumor growth in mouse xenografts	Ref. [31]
C15:1	Human lung A549 and H1299	Inhibit viability, invasion, and migration and TGF-β-induced epithelial–mesenchymal transition (EMT) by inactivating PI3K/Akt/mTOR signaling pathway	Ref. [35]
C15:1	Human colon SW480	Cause G0/G1 phase cell arrest, trigger intrinsic apoptosis and autophagy modulated by ROS generation	Ref. [36]
C15:1	Human endometrial Ishikawa and HEC-1-B	Induce apoptosis and autophagy via inhibiting PI3K/Akt/mTOR pathway in vivo and in vitro; inhibit tumor growth in mouse xenografts	Ref. [37]
C17:1	Human liver hepatoblastoma HepG2	Suppress viability, migration, and invasion by inhibiting the activation of the mitogen-activated protein kinase/MMP, Rho/Rho-associated protein kinase and PI3K/Akt signaling pathways; inhibit tumor growth in mouse xenografts	Ref. [38]
C17:1	Human liver hepatoblastoma HepG2	Inhibit cisplatin-induced autophagy via AMPK/ULK1 signaling and increase cisplatin-induced apoptosis via the PI3K/Akt/mTOR pathway	Ref. [39]
C17:1	Human liver hepatoblastoma HepG2	Inhibit cisplatin-induced autophagy via AMPK/ULK1 signaling and increase cisplatin-induced apoptosis via the PI3K/Akt/mTOR pathway in cancer cells, activate autophagy and reverse cisplatin-induced apoptosis in normal hepatocytes; synergistic cancer suppression with cisplatin	Ref. [43]

## Data Availability

Data is contained within the article.
